# Analysis of key microRNA molecules associated with acute kidney injury based on bioinformatics method

**DOI:** 10.1097/MD.0000000000041785

**Published:** 2025-03-07

**Authors:** Dongzhi Liu, Xiaoyang Zhang, Jialin Xu, Chuang Chen, Hongyi Shao, Xingxiang Chen, Dayong Wu, Qiang Ma, Wenmin Wang, Yan Wang

**Affiliations:** a Department of Critical Care Medicine, Shaoxing Central Hospital, Shaoxing, Zhejiang, China; b The Yangtze River Delta Biological Medicine Research and Development Center of Zhejiang Province, Yangtze Delta Region Institution of Tsinghua University, Hangzhou, Zhejiang, China.

**Keywords:** acute kidney injury, bioinformatics, microRNAs

## Abstract

**Rationale:**

Acute kidney injury (AKI) is a critical condition with limited early detection biomarkers and therapeutic options. This study aims to identify differentially expressed genes and potential microRNAs (miRNAs) as detection and therapeutic targets for AKI using bioinformatics-based analysis.

**Patient concerns:**

The study focuses on AKI as a major health concern with a need for improved biomarkers to monitor and treat this condition effectively.

**Diagnoses:**

The bioinformatics analysis was conducted on the Gene Expression Omnibus database to identify key differentially expressed genes related to AKI. Additionally, potential miRNAs associated with these genes were predicted to provide further insight into AKI diagnosis and therapeutic strategies.

**Interventions:**

Raw chip data from the Gene Expression Omnibus database were analyzed using coexpression complex analysis of weighted genes to identify differentially expressed genes associated with AKI. Gene set enrichment analysis and gene ontology analyses were performed to examine the pathways involved. A gene-miRNA regulatory network was constructed to explore potential therapeutic targets.

**Outcomes:**

A total of 277 differentially expressed genes were identified, with 200 genes upregulated and 77 downregulated. Significant enrichment pathways included neuroactive ligand-receptor interactions, Leishmania infection, prion disease, and electrocardiogram receptor interactions. Key enriched pathways from the Kyoto Encyclopedia of Genes and Genomes included the cytokine receptor binding pathway, chemokine signaling pathway, phosphatidylinositol-3-kinase/protein kinase B signaling pathway, and nuclear transcription factor kappa B signaling pathway. Ten hub genes, namely intercellular adhesion molecule 1 (*ICAM1*), C-X-C chemokine ligand 8 (*CXCL8*), toll-like receptor 2 (*TLR2*), selectin L (*SELL*), cytotoxic T lymphocyte-associated antigen (*CTLA4*), cell differentiation antigen 69 (*CD69*), disaccharide proteoglycan (*BGN*), C-X-C chemokine ligand 13 (*CXCL13*), metalloproteinase inhibitor 1 (*TIMP1*), and chemokine receptor 4 (*CXCR4*), were identified. Twelve critical miRNAs, namely hsa-miR-335-5p, hsa-miR-92a-3p, hsa-miR-146a-5p, hsa-miR-155-5p, hsa-miR-4426, hsa-miR-26b-5p, hsa-miR-4462b, hsa-miR-4647, hsa-miR-32-5p, hsa-miR-92b-3p, hsa-miR-98-5p, and hsa-miR-93-5p, were also identified.

**Lessons:**

This bioinformatics analysis highlights 277 differentially expressed genes and 12 potential miRNAs that may serve as biomarkers for AKI detection and therapy. These findings contribute to a better understanding of the molecular mechanisms underlying AKI and offer promising targets for future diagnostic and therapeutic strategies.

## 1. Introduction

Acute kidney injury (AKI) refers to rapid functional impairment of the kidney in a short period, resulting in disturbance of metabolites water and electrolytes in the body, which can be life-threatening in severe cases.^[1,2]^ Common causes include low blood pressure, heart failure, kidney vasculopathy, and drug poisoning.^[3,4]^

The clinical manifestations of AKI include decreased urine volume, light-colored urine, edema, nausea and vomiting, and fatigue. Diagnosis requires a variety of tests, including urine, blood and imaging, to determine whether kidney function is impaired. In terms of treatment, it is first necessary to treat the cause, such as correcting low blood pressure and controlling heart failure.^[5–7]^ Supportive treatment is also required, including fluid rehydration, correcting electrolyte disturbances, and maintaining acid-base balance. Severe AKI and kidney replacement therapy patients, like dialysis or transplantation of a kidney, may be required.^[8,9]^ Prevention of AKI is also very important, and one should pay attention to avoiding the use of nephrotoxic drugs and maintain a good diet and lifestyle.^[9,10]^

It has been found that microRNAs (miRNAs) can be involved in regulating various physiological processes. Above 60% of human protein-coding genes comprise at least 1 conserved binding site of miRNA. miRNAs are significantly related to a variety of diseases, such as AKI.^[11,12]^

In the current study, co-expression complex analysis of weighted genes and differential analysis was performed on the raw chip data of healthy individuals and AKI patients in the Gene Expression Omnibus (GEO) database to obtain differentially expressed genes, perform enrichment analysis on them, and predict potential miRNAs associated with differentially expressed gene based on key genes.

## 2. Materials and methods

### 2.1. Data sources

With “AKI” and “AKI clinic” as keywords, Restrict Entrytype to “Series” and Top Organisms to “Homo sapiens,” and obtain GSE67401 and GSE126805 gene chip data as research objects after screening through NCBI’s GEO database (https://www.ncbi.nlm.nih.gov/geo/). GSE67401 consists of 368 samples. After screening for diseases and phenotypic data, 99 samples in the untreated group and 156 samples in the AKI group were finally included. GSE126805 contained 28 samples, including 12 in the untreated group and 16 in the AKI group, all of which were included in the study.

### 2.2. Data preprocessing and screening of differentially expressed genes

ID conversion of GSE67401 data was performed using biodbnet (https://biodbnet-abcc.ncifcrf.gov/) for subsequent difference analysis. The ID transformation of GSE126805 data is carried out through the dplyr package (V 1.1.4) in R software (V 4.3.2), and the corresponding gene expression matrix file is obtained. For the gene expression matrix file of GSE67401, differential gene screening was performed using the R software limma package (V 3.58.0). The differential gene screening criteria were |logFC| > 1 and the adjusted *P* value < .05.

### 2.3. Gene set enrichment analysis (GSEA)

In this present study, we downloaded the c2.Cp.Kegg.V2023.1.Hs.Symbols.GMT collections from the Molecular Signatures Database (https://www.gsea-msigdb.org/gsea/msigdb). This gene set collection contains Kyoto Encyclopedia of Genes and Genomes (KEGG) pathway gene sets relevant to Homo sapiens, which were used to explore the biological pathways involved in AKI. To ensure the reliability and representativeness of the analysis, we set the minimum size of the gene set to 15 and the maximum size to 500. Gene sets smaller than 15 genes might be too specific or lack statistical power, while gene sets larger than 500 genes could be too broad and might not accurately reflect the underlying biological processes (BP). We performed 1000 permutations of the gene expression data. Permutation is a statistical method used to assess the significance of the enrichment results. By randomly shuffling the gene-phenotype associations multiple times, we can estimate the probability that the observed enrichment is due to chance. For the enrichment results, we considered a *P* value of < .05 and a false discovery rate < 0.25 as significant. The *P* value measures the probability of obtaining the observed enrichment or a more extreme result under the null hypothesis (no real enrichment). The false discovery rate is a method for controlling the proportion of false positives among the significant results, and setting it to < 0.25 helps balance the discovery of true-positive enrichments and the control of false-positive findings.

### 2.4. Functional enrichment analysis of intersection genes

First, differential genes and genes in gene modules were introduced into Venny 2.1.0 for intersection analysis to obtain intersection genes. Functional enrichment analyses of these intersected genes were done by using the R software ClusterProfiler package, including gene ontology (GO) and KEGG pathway analyses. GO functional analysis such as cell components, BP, and molecular functions. *P* value of < .05 was regarded as a screening criterion to identify significantly enriched gene set entries.

### 2.5. Construction of protein binding complex

In contemplation to reveal the role of AKI-related proteins and their mutual relations, they will overlap between genes in the online database STRING (V 11.5) (HTTPS://cn.string-db.org/), to construct the protein binding complex. During the build process, set confidence > 0.4 as the screening criteria. The constructed protein-protein interaction (PPI) network is then visualized using Cytoscape software (V 3.11.2). By using the degree ranking in the CytoNCA plug-in (V 2.3.0), the top 10 genes are chosen as hub genes.

### 2.6. Hub gene-associated miRNA

Using the Network Analyst (V 3.0) (https://www.networkanalyst.ca/) online platform, the obtained 10 hub genes were manipulated to their corresponding miRNAs to identify the miRNA-gene binding in the gene regulatory complex. Hence, the miRNA-gene complex map was visualized by Cytoscape software.

## 3. Results

### 3.1. Screening results of differential genes

**Figure 1.**
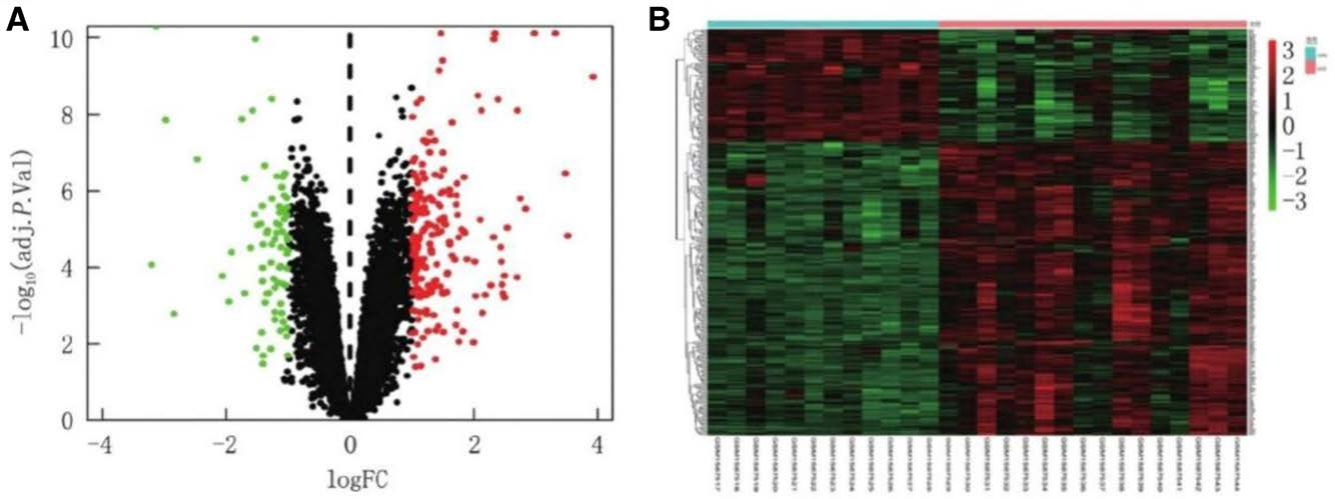
Volcanic and heat maps of differentially expressed genes in GSE67401. (A) In the volcano plot, genes are plotted based on their fold change (logFC) and *P* value. The thresholds for identifying differentially expressed genes are set as |logFC| > 1 and *P* < .05. Genes meeting these criteria are considered significantly differentially expressed. (B) The heat map visualizes the expression levels of differentially expressed genes. Red represents upregulated genes, indicating that their expression levels are higher in the acute kidney injury (AKI) group compared to the untreated group. Green represents downregulated genes, meaning their expression levels are lower in the AKI group. Black indicates genes with intermediate expression levels. The intensity of the color corresponds to the degree of expression change; the more significant the gene expression difference, the darker the color.

According to the gene expression data of GSE67401, the untreated group and the AKI group were compared. To screen for differential genes and visualize them, use the ggplot2 package in R software. |log FC| > 1 and *P* < .05 were the criteria for screening. Finally, after data processing, 277 differentially acting genes were collected, of which 200 genes were up-controlled and 77 genes were down-controlled. Detailed genetic heat maps and volcanic maps are shown in Figure 1. In the heat map, red indicates up-controlled genes, green indicates down-controlled genes, and black indicates intermediate expression levels. The more significant the gene expression, the darker the color.

### 3.2. The result of GSEA analysis

**Figure 2.**
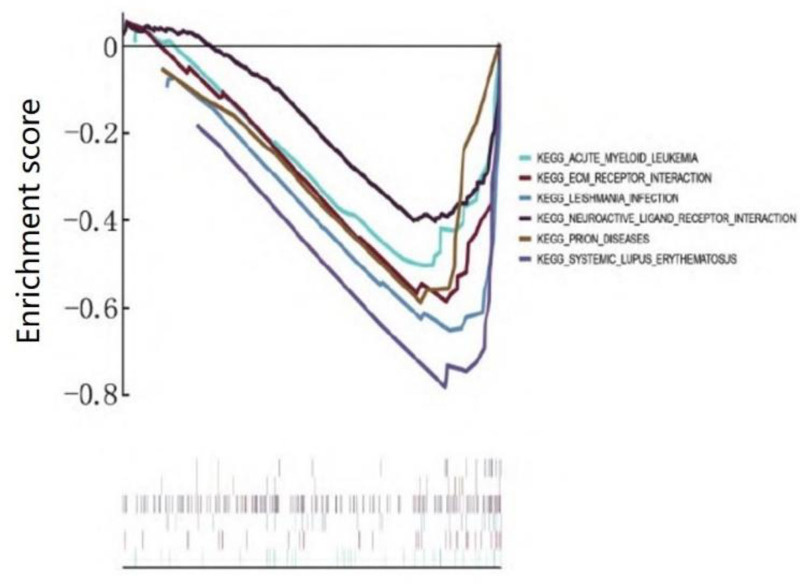
Gene set enrichment analysis. This figure presents the results of the Gene Set Enrichment Analysis (GSEA) conducted on the expression matrix of all genes in the GSE67401 data. The analysis aimed to identify the major enrichment pathways associated with acute kidney injury. The enrichment pathways primarily occur in the neuroactive ligand-receptor interaction pathway, leishmania infection, prion disease, and electrocardiogram receptor interaction pathway. The significance of these pathways was determined by setting thresholds for *P* values < .05 and false discovery rate < 0.25. Each pathway shown in the figure represents a biological process or molecular mechanism that is potentially involved in the development or progression of acute kidney injury. KEGG = Kyoto Encyclopedia of Genes and Genomes.

Through the GSEA analysis of the expression matrix of all genes in the GSE67401 data, the results unveiled the enrichment pathway primarily takes place in the neuroactive ligand-receptor interaction pathway, leishmania infection, prion disease, and electrocardiogram receptor interaction pathway, as shown in Figure 2.

### 3.3. Results of intersection genes enrichment analysis

**Figure 3.**
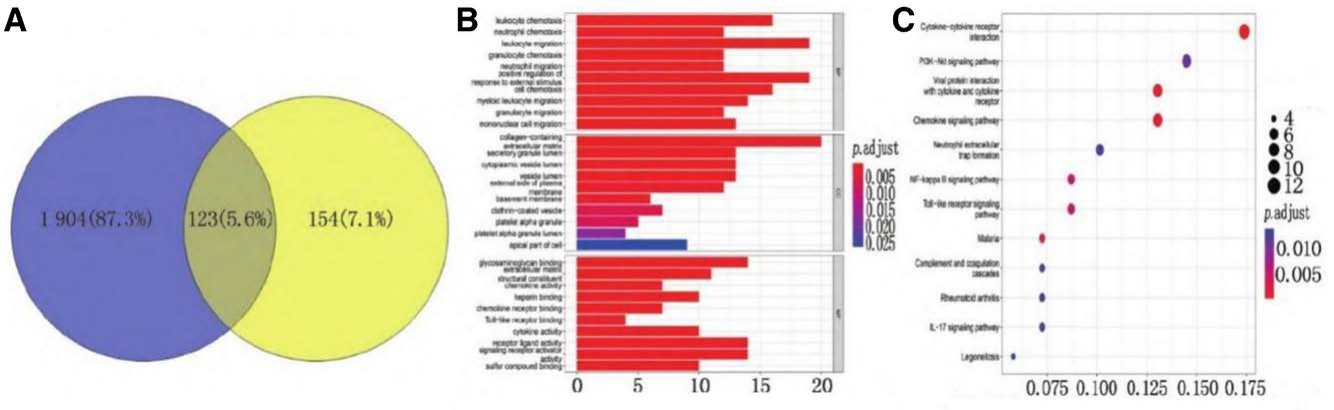
Intersection gene Venn diagram and GO and KEGG enrichment analysis. (A) Venn diagram of intersecting genes: this Venn diagram shows the intersection of the 277 differential genes obtained from the analysis of GSE67401 data and the 2027 genes in gene modules. (B) Gene ontology (GO) enrichment analysis of intersection genes: the GO enrichment analysis of the intersection genes was performed using the R software ClusterProfiler package. The results are presented here. For biological processes (BP), the enriched items are mainly concentrated in processes such as the migration of leukocytes, positive regulatory response to external stimuli, leukocyte chemotaxis, migration of myeloid leukocyte, monocyte migration, and neutrophil chemotaxis. In terms of cell components (CC), the items are mainly related to collagen, extracellular matrix secretory granule cavity, cytoplasmic vesicle cavity, plasma vesicle lateral cavity, and membrane apical part. Regarding molecular functions (MF), the enrichment is mainly on the chemokine activity of structural components, heparin-binding, chemokine receptor binding, toll-like receptor binding, and cytokine activity. A *P* value < .05 was used as the screening criterion to identify significantly enriched gene set entries. (C). Kyoto Encyclopedia of Genes and Genomes (KEGG) enrichment analysis of intersection genes: the KEGG enrichment analysis of the intersection genes was also carried out using the R software ClusterProfiler package.

GSE67401 and GSE126805 gene expression data sets in the GEO database were used for analysis. The obtained 277 differential genes and 2027 genes in gene modules were imported into Venny 2.1.0 for intersection, and 123 intersection genes were obtained (Fig. 3A). There were 114 up-controlled genes and 9 down-controlled genes. The R software Cluster Profiler package was used for GO function enrichment analysis and KEGG pathway of the obtained intersection genes, and *P* < .05 was regarded as the criteria for enrichment item screening.

GO enrichment results (Fig. 3B) showed that items related to BP were mainly concentrated in the migration of leukocytes, positive regulatory response to external stimuli, leukocyte chemotaxis, migration of myeloid leukocytes, monocyte migration, neutrophil chemotaxis, etc. The items related to cell components were mainly concentrated in collagen, extracellular matrix secretory granule cavity, cytoplasmic vesicle cavity, plasma vesicle lateral cavity, and membrane apical part. The articles related to molecular functions were mainly concentrated on the chemokine activity of structural components, heparin-binding, chemokine receptor binding, toll-like receptor binding, and cytokine activity. The KEGG enrichment analysis (Fig. 3C) results show that the first 12 pathways are mainly concentrated in cytokine receptor binding, chemokine signaling pathway, nuclear transcription factor kappa B signaling pathway, phosphatidylinositol-3 kinase/protein kinase B (PI3K-AKT) signaling pathway, interleukin-17 signaling pathway, toll-like receptor signaling pathway, and other related processes.

### 3.4. Screening results of PPI complex and hub gene

**Figure 4.**
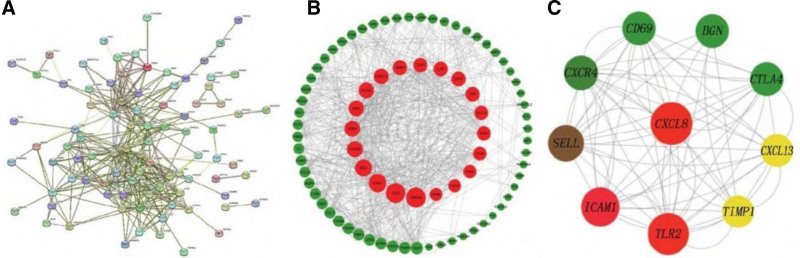
Protein-protein interaction (PPI) complex map and hub gene complex. (A) PPI complex map: the intersection genes were imported into the String online database to construct the PPI complex. This map represents the interactions between acute kidney injury (AKI)-related proteins. The confidence level was set > 0.4 as the screening criterion during the construction process. (B) PPI complex diagram after Cytoscape visualization: the PPI network obtained from the String database was visualized using Cytoscape software. (C) Hub gene complex: based on the degree ranking calculated by the CytoNCA plug-in in Cytoscape, the top 10 genes were selected as hub genes. These hub genes are C-X-C chemokine ligand 8 (*CXCL8*), toll-like receptor 2 (*TLR2*), intercellular adhesion molecule 1 (ICAM1), selectin L (SELL), chemokine receptor 4 (*CXCR4*), cytotoxic T lymphocyte-associated antigen (*CTLA4*), cell differentiation antigen 69 (*CD69*), disaccharide proteoglycan (*BGN*), C-X-C chemokine ligand 13 (*CXCL13*), and metalloproteinase inhibitor 1 (*TIMP1*). The hub gene complex shows the relationships between these key genes, which are likely to play important roles in the incidence and development of acute kidney injury.

The intersection gene was imported into the String online database to collect the PPI complex (Fig. 4A). Then, Cytoscape software is used to visualize the obtained PPI network (Fig. 4B), and the CytoNCA plug-in is used to calculate the degree value of the node. Increasing the degree value, the larger the node. The top 10 genes were chosen as hub genes. They are C-X-C chemokine ligand 8 (*CXCL8*), toll-like receptor 2 (*TLR2*), intercellular adhesion molecule 1 (*ICAM1*), selectin L (*SELL*), chemokine receptor 4 (*CXCR4*), cytotoxic T lymphocyte-associated antigen (*CTLA4*), cell differentiation antigen 69 (CD69), disaccharide proteoglycan (*BGN*), C-X-C chemokine ligand 13 (*CXCL13*), and metalloproteinase inhibitor 1 (*TIMP1*). The hub gene network was established for these 10 key genes (Fig. 4C).

### 3.5. miRNAs-hub gene regulatory complex was established

**Figure 5.**
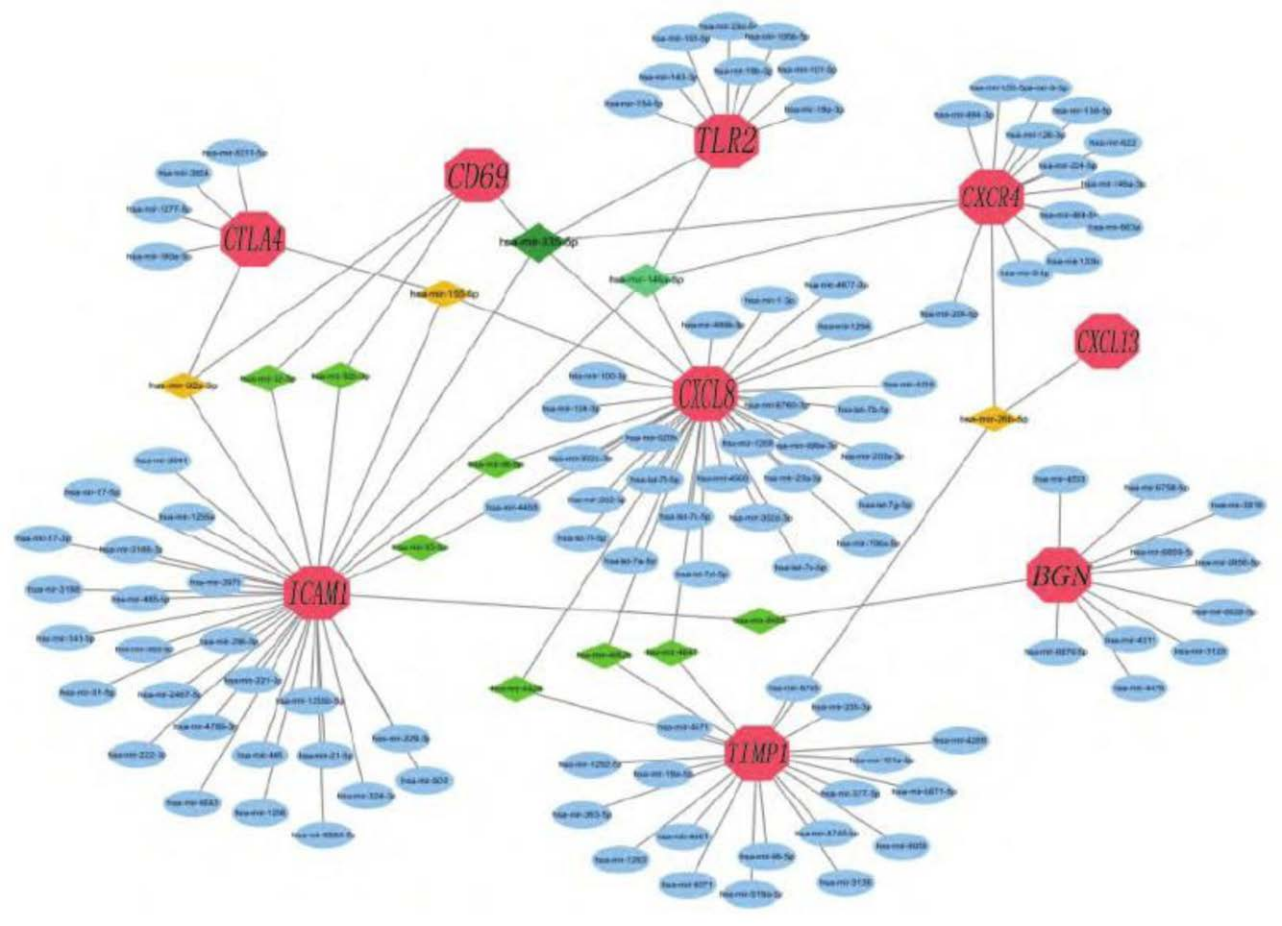
miRNAs-hub gene regulatory network. This figure depicts the miRNA-hub gene regulatory complex established using Cytoscape based on the Network Analyst dataset. miRNAs play a crucial role in regulating gene expression. In this network, each miRNA is connected to its corresponding target hub genes. For example, miRNA (hsa-miR-335-5p) has 5 target genes (*CXCL8*, *TLR2*, *ICAM1*, *CXCR4*, and *CD69*), miRNA (hsa-miR-146a-5p) has 4 target genes (*CXCL8*, *TLR2*, *ICAM1*, and *CXCR4*), and so on. The connections represent the regulatory relationships between miRNAs and genes, providing insights into the molecular mechanisms underlying the pathogenesis of acute kidney injury at the post-transcriptional level.

miRNA plays multiple parts in the control of gene expression. According to the Network Analyst dataset, the miRNA-hub gene regulatory complex was established using Cytoscape. The hub gene and its corresponding regulatory miRNAs are shown in Figure 5. Among them, miRNA (hsa-miR-335-5p) had 5 target genes (*CXCL8*, *TLR2*, *ICAM1*, *CXCR4*, and *CD69*). miRNA (hsa-miR-146a-5p) has 4 target genes (*CXCL8*, *TLR2*, *ICAM1*, and *CXCR4*). miRNA (hsa-miR92a-3p) has 3 target factors (*ICAM1*, *CTLA4*, and *CD69*). miRNA (hsa-miR-155-5p) has 3 target genes (*CXCL8*, *ICAM1*, and *CTLA4*). miRNA (hsa-miR-26b-5p) had 3 target genes (*CTLA4*, *CXCL13*, and *TIMP1*). In addition, *CXCL8* and *ICAM1* were the main targets of the 3 miRNAs (hsa-miR-4426, hsa-miR-4462b, and hsa-miR-4647). ICAM1 and CD69 are the main targets of 2 miRNAs (hsa-miR-32-5p and hsa-miR-92b-3p). CXCL8 and ICAM1 are the main targets of 2 miRNAs (hsa-miR-98-5p and hsa-miR-93-5p).

## 4. Discussion

AKI is a sudden onset of abnormal kidney function, usually manifested by increased blood creatinine levels and decreased urine output. It occurs due to a variety of causes, including diseases of the kidneys themselves, the use of drugs or poisons, infections, trauma, or other serious illnesses.^[13,14]^ Under normal circumstances, waste and excess water were filtered from the blood and expelled from the body by the kidneys. However, when the kidney is damaged, it may not be able to complete this process effectively, causing waste products and water from the blood to accumulate in the body, which can cause a range of symptoms and complications.^[15–17]^

Symptoms of AKI include edema, hypertension, nausea, vomiting, fatigue, and loss of appetite. If left untreated, severe AKI can lead to complications such as uremia, electrolyte disturbance, and anemia, which can even be life-threatening.^[18,19]^ Therefore, patients with symptoms of AKI should seek medical attention as soon as possible for diagnosis and treatment. At the same time, preventive measures are also very important, such as avoiding the use of drugs that are harmful to the kidney, maintaining a good diet, and getting regular medical checkups.^[19–21]^

In this study, GSE67401 and GSE126805 gene expression datasets in the GEO database were used for analysis. First, 123 overlapping genes were obtained by using weighted gene co-expression network analysis and difference analysis. Among them, 117 genes were up-controlled and 9 genes were down-controlled. Then the functional enrichment analysis of these intersection genes was performed, and a protein binding complex was established. This process screened out 10 key genes, including *CXCL8*, *TLR2*, *ICAM1*, *SELL*, and *CXCL4*, which may play an important part in the incidence and growth of AKI. The results of GO and KEGG functional enrichment analysis showed that the core genes were closely related to leukocyte migration, interleukin, chemokine activity, and collagen. The signaling pathways involved include PI3K-AKT signaling pathway, toll-like receptor signaling pathway, nuclear transcription factor kappa B signaling pathway, and interleukin-17 signaling pathway. These pathways are nearly associated with the stimulation of inflammation-related signals and the release of pro-inflammatory factors, suggesting that they play a significant part in the pathogenesis and exacerbation of AKI. Migration of leukocytes takes place in the occurrence of AKI, thus arresting of leukocyte migration is a major strategy for controlling the disease and alleviating symptoms. Studies have shown that the abnormality of the PI3K-AKT cell signaling pathway is believed to be related to the pathogenesis of AKI and has a great impact on apoptosis and inflammation induced by AKI, as well as chronic renal insufficiency induced by long-term AKI.^[21,22]^ The GO and KEGG enrichment pathway analysis results indicate that the key genes detected in this study may take place in the occurrence of AKI via the above pathways.

miRNAs can control the human gene’s expression and take place a significant part in the pathogenesis of autoimmune diseases.^[23,24]^ Relevant studies have found and verified that the increase of long-chain noncoding RNA MEG3 can inhibit the up-control of miR-98-5p in AKI rats, and promote the expression of interleukin-10 by regulating miR-98-5p.^[24–26]^ Other studies have found that miR-146a-5p reduces the damage of renal mucosa by upregulating ring finger protein 8 and inhibiting Notch1/mTORC1 pathway.^[26,27]^ Some scholars have found that inflammatory mediators induce the miR-155 expression in the renal tissue fibroblasts of AKI patients by downregulating the expression of cytokine signaling suppressor protein 1.^[28]^ It was found that toll-like receptor 4 is directly downstream of hsa-miR-375, and its level is negatively mediated by has-miR-375. hsa-miR-146a-5p, has-miR-155-5p, hsa-miR-335-5p, hsa-miR-32-5p, hsa-miR-98-5p, hsa-miR-93-5p, and their target genes have been reported to be associated with AKI.^[29,30]^ Other miRNAs obtained in this study included hsa-miR-92a-3p, hsa-miR-26b-5p, hsa-miR-4426, hsa-miR-4462b, hsa-miR-4647, and hsa-miR-92b-3p. And its target genes may take place as part of AKI.

In this study, we identified 12 potentially critical miRNAs associated with AKI through bioinformatics analysis. Comparing our findings with previous studies on AKI-related miRNAs provides valuable insights. Jiao et al^[31]^ reported that exosomal miR-30d-5p from neutrophils contributes to sepsis-related acute lung injury by modulating macrophage functions. While our study focused on AKI, the involvement of miR-30d-5p in inflammation-related processes in their research aligns with our finding that some of our identified miRNAs, such as hsa-miR-146a-5p and hsa-miR-155-5p, are also related to inflammation. This suggests a possible common inflammatory-regulatory mechanism in different sepsis-related organ injuries. However, the specific regulatory roles of these miRNAs in AKI may differ from those in acute lung injury, and further research is required to clarify these distinctions. Yuan et al found that miR-195a-5p in autophagy-deficient macrophages impairs mitochondrial function in tubular epithelial cells, exacerbating AKI in mice. Although our study did not identify miR-195a-5p,^[32]^ we identified miRNAs like hsa-miR-335-5p with target genes potentially involved in cellular homeostasis-related processes, which may be related to mitochondrial function. This indicates that different miRNAs might converge on similar cellular mechanisms to affect AKI development, but the detailed regulatory relationships are yet to be fully explored. Tyson et al^[33]^ identified miR-6826-5p and miR-6811-3p as potential biomarkers for predicting AKI in severe alcohol-related hepatitis. Our study aimed to find miRNAs associated with AKI in a more general context. While their findings were specific to alcohol-related hepatitis, our identified miRNAs may have broader applications in AKI prediction. Additionally, our miRNAs could potentially complement theirs. For example, they may be more effective in predicting AKI in non-alcohol-related cases. Future research could explore the combined use of these miRNAs to improve the accuracy of AKI prediction. Cui et al^[34]^ demonstrated that lncRNA GAS6-AS2 regulates the miR-136-5p/OXSR1 axis to alleviate sepsis-related AKI. Our study did not directly involve lncRNA-miRNA interactions. However, the role of miR-136-5p in their research suggests that there could be complex regulatory networks in AKI that our identified miRNAs may be a part of. Future studies could investigate whether our miRNAs interact with lncRNAs in a similar way, which would expand our understanding of the molecular mechanisms underlying AKI. In conclusion, our study adds to the growing knowledge of AKI-related miRNAs. Comparing our results with previous studies reveals both similarities and differences in miRNA functions and regulatory mechanisms. These comparisons emphasize the complexity of miRNA-mediated regulation in AKI and offer directions for future research to better understand the roles of these miRNAs as therapeutic targets or biomarkers.

Weighted gene co-expression network analysis was employed to probe differential genes, and combined with the GSEA gene set enrichment method and the gene-miRNA regulatory network, it was established to explore the potential regulatory mechanisms of AKI and identify new potential indicators and pathways, which is innovative. The gene-miRNA regulatory complex plays a significant part in the pathophysiology of AKI. The shortcoming of this study is that it only analyzed the microarray expression profile through bioinformatics but did not verify it through relevant experiments and explore the detailed mechanism of hub gene and miRNA regulation of AKI. Therefore, it is necessary to expand clinical research samples and further experiments to clarify the mechanism.

However, this study has several limitations. First, our analysis was solely based on bioinformatics methods, relying on publicly available gene expression data from the GEO database. The lack of experimental validation, such as in vitro or in vivo studies, means that the identified key genes and miRNAs remain theoretical. Their actual roles in the development and progression of AKI have not been directly verified. Second, the gene-miRNA regulatory relationships predicted in this study were based on bioinformatics algorithms. Although these algorithms have certain predictive power, there may be false positives or false negatives. The actual regulatory mechanisms may be more complex than what we have predicted. Third, the datasets used in this study had a relatively limited sample size, especially in the GSE126805 dataset. A larger sample size would be beneficial for more accurate identification of differentially expressed genes and more reliable determination of their relationships with AKI. Future research should focus on validating these findings through experimental studies and expanding the sample size to further clarify the role of these genes and miRNAs in AKI.

## 5. Conclusion

In the current study, overall 277 differentially acting genes were screened for possible involvement in the incidence and growth of AKI, and 10 pivotal genes and 12 miRNAs were identified as biomarkers for AKI.

## Author contributions

**Conceptualization:** Dongzhi Liu, Xiaoyang Zhang, Chuang Chen, Yan Wang.

**Writing – original draft:** Dongzhi Liu, Jialin Xu, Chuang Chen, Qiang Ma, Wenmin Wang.

**Writing – review & editing:** Dongzhi Liu, Xiaoyang Zhang, Dayong Wu.

**Methodology:** Xiaoyang Zhang.

**Data curation:** Jialin Xu, Hongyi Shao.

**Validation:** Jialin Xu, Dayong Wu, Wenmin Wang.

**Formal analysis:** Chuang Chen.

**Investigation:** Hongyi Shao, Xingxiang Chen, Qiang Ma, Wenmin Wang.

**Resources:** Xingxiang Chen, Yan Wang.

**Software:** Xingxiang Chen.

**Funding acquisition:** Dayong Wu.

**Visualization:** Qiang Ma.

**Supervision:** Yan Wang.
